# In Vivo 3D Liver Imaging at 7 T Using kT‐Point pTx Pulses and a 32‐Tx‐Channel Whole‐Body Radiofrequency Coil Array

**DOI:** 10.1002/nbm.70170

**Published:** 2025-10-28

**Authors:** Johannes A. Grimm, Christoph S. Aigner, Sebastian Dietrich, Stephan Orzada, Thomas M. Fiedler, Simon Schmidt, Constantin Schorling, Harald H. Quick, Armin M. Nagel, Mark E. Ladd, Sebastian Schmitter

**Affiliations:** ^1^ Medical Physics in Radiology German Cancer Research Center (DKFZ) Heidelberg Germany; ^2^ Faculty of Physics and Astronomy Heidelberg University Heidelberg Germany; ^3^ Physikalisch‐Technische Bundesanstalt (PTB) Braunschweig and Berlin Germany; ^4^ Max Planck Research Group MR Physics Max Planck Institute for Human Development Berlin Germany; ^5^ Erwin L. Hahn Institute for Magnetic Resonance Imaging University Duisburg‐Essen Essen Germany; ^6^ High‐Field and Hybrid MR Imaging University Hospital Essen Essen Germany; ^7^ Institute of Radiology University Hospital Erlangen, Friedrich‐Alexander‐Universität Erlangen‐Nürnberg (FAU) Erlangen‐Nürnberg Germany; ^8^ Faculty of Medicine Heidelberg University Heidelberg Germany; ^9^ Center for Magnetic Resonance Research University of Minnesota Minneapolis Minnesota USA

## Abstract

At 7 T, a main challenge is the flip angle variations arising from the spatially inhomogeneous *B*_1_^+^ transmit profiles of the RF coils. To address this problem, static pTx with local pTx body arrays has been used. However, for larger organs, such as the liver, local arrays provide insufficient homogeneity, and often dynamic pTx is needed. In this study, the benefits of a 32‐Tx‐channel remote array are shown. Relative channel‐wise 3D *B*_1_^+^ maps are acquired in free breathing in 11:38 min with a radial phase encoding (RPE) acquisition scheme in five healthy volunteers at 7 T. For each subject, an ROI is drawn manually covering the whole liver. The necessary number of k_T_‐point pTx pulses is analyzed by comparing L‐curves that depict the tradeoff between FA homogeneity and RF power for a large range of regularization parameters. For all subjects, no FA dropouts remain, and CV values under 17% can be achieved with magnitude and phase shimming. Two to three k_T_‐points showed a good tradeoff between FA homogeneity and RF power. To analyze different channel numbers, a fixed phase between the channel‐wise *B*_1_^+^ maps is set to achieve 8, 16, or 20 *B*_1_^+^ maps for shimming. The higher channel number showed superior shimming performance for static and dynamic pTx. Comparing 32 versus 8 individual channels, a 1.3‐ to 1.6‐fold improvement in homogeneity can be found for the same RF power using phase shimming. With 32 Tx channels, static pTx shim and two k_T_‐points were applied in vivo in free breathing with a 3D RPE GRE sequence with 1.4‐mm isotropic resolution. The in vivo signal magnitudes of the small FA acquisitions showed good agreement with the predicted relative *B*_1_^+^ maps while neglecting *B*_0_ nonuniformity. In summary, this study shows the advantages of using a remote 32‐Tx channel array for in vivo liver imaging at 7 T.

## Graphical abstract

Using a remote 32‐Tx channel coil array static pTx yielded sufficient flip angle (FA) homogeneity in the entire liver in normal BMI subjects. With two k_T_‐points, the homogeneity is improved further to achieve a FA coefficient of variation of under 10% in all subjects.

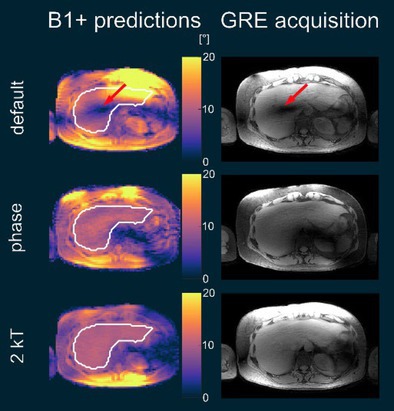

**Abbreviations**
CV: Coefficient of variation
FA: Flip angle
FoV: Field of view
GRE: Gradient recalled echo
pTx: Parallel transmit
RF: Radiofrequency
ROI: Region of interest
RPE: Radial phase encoding
SAR: Specific absorption rate
SNR: Signal‐to‐noise ratio
TIAMO: Time‐interleaved acquisition of modes
Tx: Transmit
UHF: Ultrahigh field
VOPs: Virtual observation points

## 1 Introduction

In ultrahigh field (UHF) body MRI, a main challenge is the presence of flip angle (FA) variations or voids that arise from the spatially inhomogeneous *B*_1_^+^ transmit profiles of the radiofrequency (RF) coils. To address this issue, several methods have been developed, including the use of dedicated coils [1], dielectric pads [2, 3], or adiabatic pulses [4].

Another method that has been successfully applied is parallel transmit (pTx), either static pTx or dynamic pTx [5, 6]. Hereby, multiple transmit (Tx) channel arrays are used for transmission, with most pTx systems being equipped with 8 Tx channels. The fields $B_{1,\mathrm{ch}}^{+}\left(r,t\right)$ of each Tx element ch of the array are weighted by a static or time‐dependent complex factor, which enables shaping the resulting superposed field in a static or dynamic manner.

Initial applications in the human body used static pTx, which is also often denoted as *B*_1_^+^ shimming [7]. Here, the individual Tx channels are fed by a common RF pulse shape that is weighted by a Tx‐channel–dependent amplitude $a_{\mathrm{ch}}$ and phase term $\phi_{\mathrm{ch}}$, which is called magnitude and phase shimming. As a result, a spatially constant superposed *B*_1_^+^ field is achieved, which is for example optimized to achieve a given *B*_1_^+^ homogeneity within the target organ or a high transmit efficiency η, which denotes the level of constructive interference between the individual *B*_1_^+^ maps [8]. In many cases in the body, only the Tx phase is adjusted in static pTx and all Tx amplitudes are set equally, which is called phase shimming. Several UHF groups showed the successful application of static pTx in the human torso, that is the prostate [8, 9], spine [10, 11], kidneys [12, 13], hip [14, 15], and breast [16], where a homogeneous *B*_1_^+^ was achieved or a high transmit efficiency.

However, when optimizing the 3D field for organs of intermediate size, such as the heart, or large organs like the liver, static pTx is typically less successful, and local dropouts of the *B*_1_^+^ field may still be observed at 7 T [17, 18, 19]. One solution is the use of the time‐interleaved acquisition of modes (TIAMO) method [20], which acquires several identical scans with two or more optimized shims with different *B*_1_^+^ shim vectors, each resulting in complementary *B*_1_^+^ dropouts, and then combines them for a single image with a more homogeneous image intensity. This has already been applied in the kidneys [21], liver [22], and also for a large pelvic region [23] and for the abdomen [24, 25].

Another possibility is the use of dynamic pTx for organs of intermediate and large size because degrees of freedom are achieved by time‐dependent complex weights $b_{\mathrm{ch}}\left(t\right)$, which are then optimized jointly with the applied excitation trajectory. For slice‐selective dynamic pTx, spokes pulses are often used, while for 3D excitation volumes, k_T_‐point pulses or SPINS pulses have been applied [26, 27, 28]. Using these techniques, dynamic pTx was already successfully applied in the human body, for example, in the heart [29, 30] and in the liver [17, 31, 32], where, for example, Runderkamp et al. [31] achieved a homogeneous excitation in the liver using five k_T_‐points and a local 8‐Tx channel array. However, an increased number of k_T_‐points tends to prolong the RF pulse because gradient blips are required between each subpulse. This longer duration can impose constraints on sequence timing and increase sensitivity to gradient imperfections. Moreover, when employing multiple subpulses, Δ*B*₀ becomes significant as the magnetization accumulates additional phase between subpulses, leading to excitation errors that worsen with more k_T_‐points. While it is possible to account for Δ*B*₀ in the optimization process [27], its inherent variability, such as that induced by respiration [33], limits the effectiveness of this approach. Thus, although using additional subpulses can enhance excitation fidelity, they also become more vulnerable to imperfections and physiological fluctuations [34] making the use of fewer subpulses advantageous.

Nevertheless, static pTx without FA dropouts for targets of intermediate or large size would be even more desirable, as static pTx does not require changing the sequence (i.e., the RF pulse waveform), it is typically less sensitive to $\Delta B_{0}$, and it is often faster to calculate compared to dynamic pTx solutions [28]. Furthermore, shorter transmission times can be achieved, and therefore potentially shorter repetition times.

A common feature of static and dynamic pTx is that both techniques use, apart from a few exceptions [24, 35], *local* transmit body coil arrays located on top/underneath the body in contrast to the (single‐ or dual‐channel) whole‐body RF transmit coils of clinical systems that are located behind the bore liner. The reasons are multifold: local transmit arrays have a higher power efficiency ($B_{1}^{+}/\surd P_{\text{input}}$) [1, 36], thus less RF power is needed, which is beneficial since most pTx systems are equipped with eight amplifiers limited to 1 or 2 kW each. Second, MR vendors do not provide a remote transmit array integrated behind the scanner bore. Installing such remote arrays would require substantial modifications to the scanner hardware, and because local receive coils are essential for achieving high signal‐to‐noise ratio (SNR), developing local Tx and Rx arrays has been the most practical solution. As a result of both arguments, local Tx arrays have so far been the more cost effective.

With newer cost‐effective amplifier modules, however, high power has become more affordable, and the focus has shifted from power efficiency to the specific absorption rate (SAR) efficiency (i.e., *B*_1_^+^ per maximum 10‐g averaged local SAR), where remote arrays are similar in SAR efficiency to local arrays for smaller excitation regions while providing a larger coverage for large excitation regions [37, 38]. Furthermore, Tx arrays are typically bulkier than Rx‐only arrays and therefore take up more space in the typically 60‐cm diameter bore of the scanner. The larger distance to the body is also beneficial because it provides more space for the elements, allowing for more Tx channels, which increases the degrees of freedom for pulse optimization and therefore the excitation fidelity for a given RF power (or SAR value). However, as the *B*_1_^+^ efficiency of the remote coil is lower than that of local coils, the higher channel count is also beneficial to counteract this effect [36]. Further benefits of whole‐body arrays are the larger field of excitation along the longitudinal direction, which is more restricted for most local body coil arrays [36], and the possibility to use local X‐nuclei transmit arrays in combination with the whole‐body proton array, for example, for large field of view (FoV) proton images for anatomic screening and, that is, sodium images for metabolic quantification [39].

Recently, a 32‐channel RF transmit system with a total of 64 kW including a remote body coil array was presented, which has been used in this paper [24]. Given the limitations of applying static pTx for intermediate and large size organs, the question arises whether more than 8 Tx channels and the larger distance to the body provided by a remote whole‐body coil array benefit the 3D FA homogenization of such organs and whether static pTx may be sufficient. For 2D slices, previous simulation studies indicated the increase in homogeneity for a complete transversal slice or a large coronal slice using dynamic spokes pTx pulses [37, 38].

This study investigates the application of static and 3D dynamic k_T_‐point pTx pulses for the whole liver in multiple subjects using a 32‐Tx‐channel whole‐body remote array. 3D free‐breathing channel‐wise relative *B*_1_^+^ maps are acquired for five subjects, and the shimming performance with static pTx and dynamic pTx k_T_‐point pulses is compared and analyzed. Furthermore, this work investigates how the number of transmit channels affects the *B*_1_^+^ shimming performance, including whether higher channel counts enable homogeneous excitations with fewer k_T_‐point subpulses.

## 2 Methods

### 2.1 Hardware

All measurements were performed on a 7 T MRI system (MAGNETOM 7 T, Siemens Healthcare, Erlangen, Germany) with a retrofitted homebuilt 32‐channel Tx whole‐body coil array located behind the bore liner [24]. The array is connected to a homebuilt 32‐channel pTx system with 2 kW peak power per channel and a 100 kHz sampling rate. In this work, two local rigid 16‐channel Rx arrays (anterior Rx array: RAPID Biomedical GmbH, Rimpar, Germany; posterior Rx array: [40] Erwin L. Hahn Institute (ELH) for MRI, Essen, Germany) are used for reception.

### 2.2 Calibration Measurements

**FIGURE 1:**
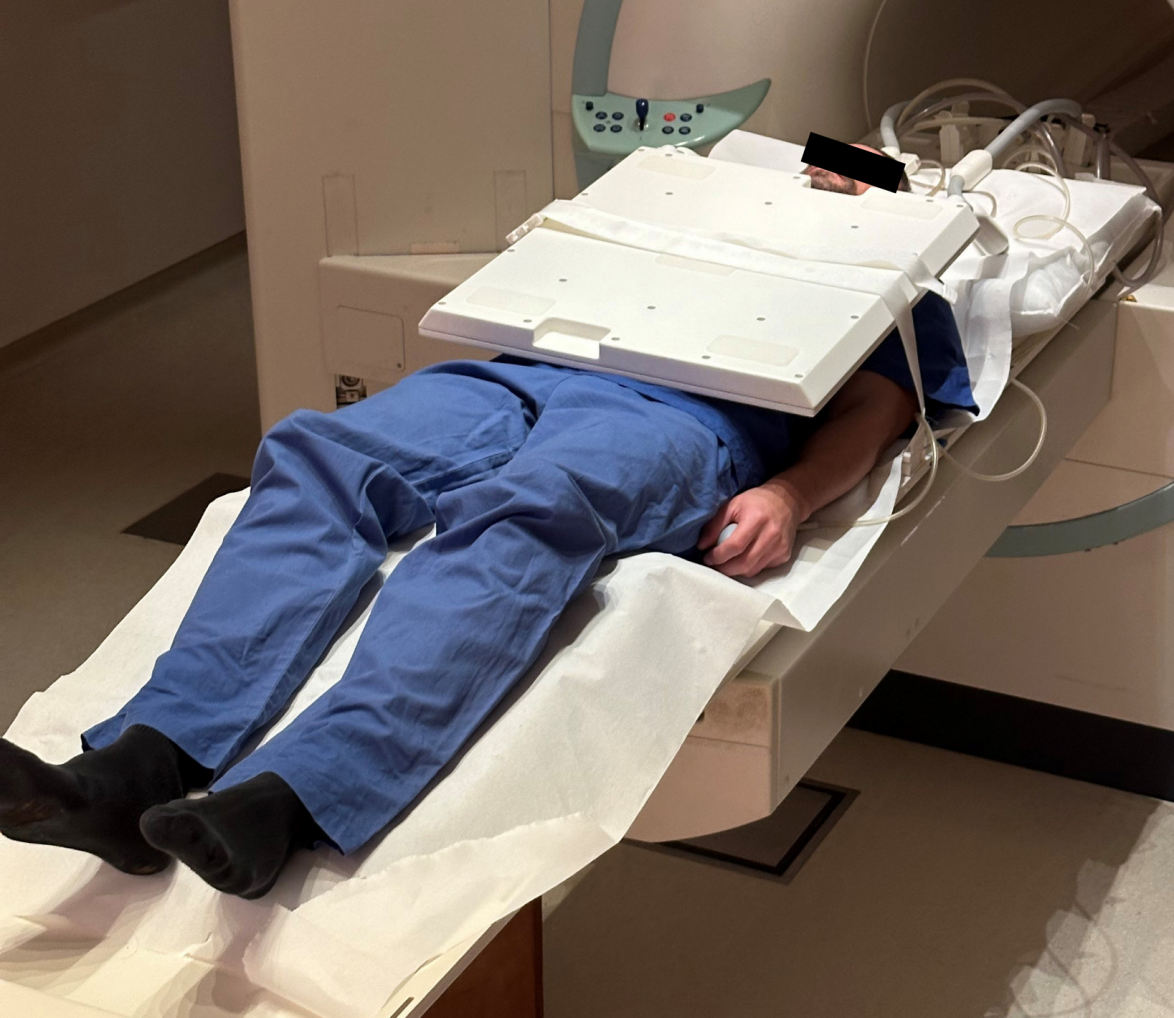
Measurement setup with two local rigid 16‐channel receive arrays. One Rx array is placed in the patient table and the second one is placed on the subject.

Five healthy volunteers (2 male and 3 female, BMI range = 19.5–26.2 kg/m^2^) were scanned in a head‐first supine position with the liver placed in the isocenter. The experimental setup is shown in Figure 1. Written informed consent according to the institutional guidelines and approval from the local ethics committee were obtained.

**FIGURE 2:**
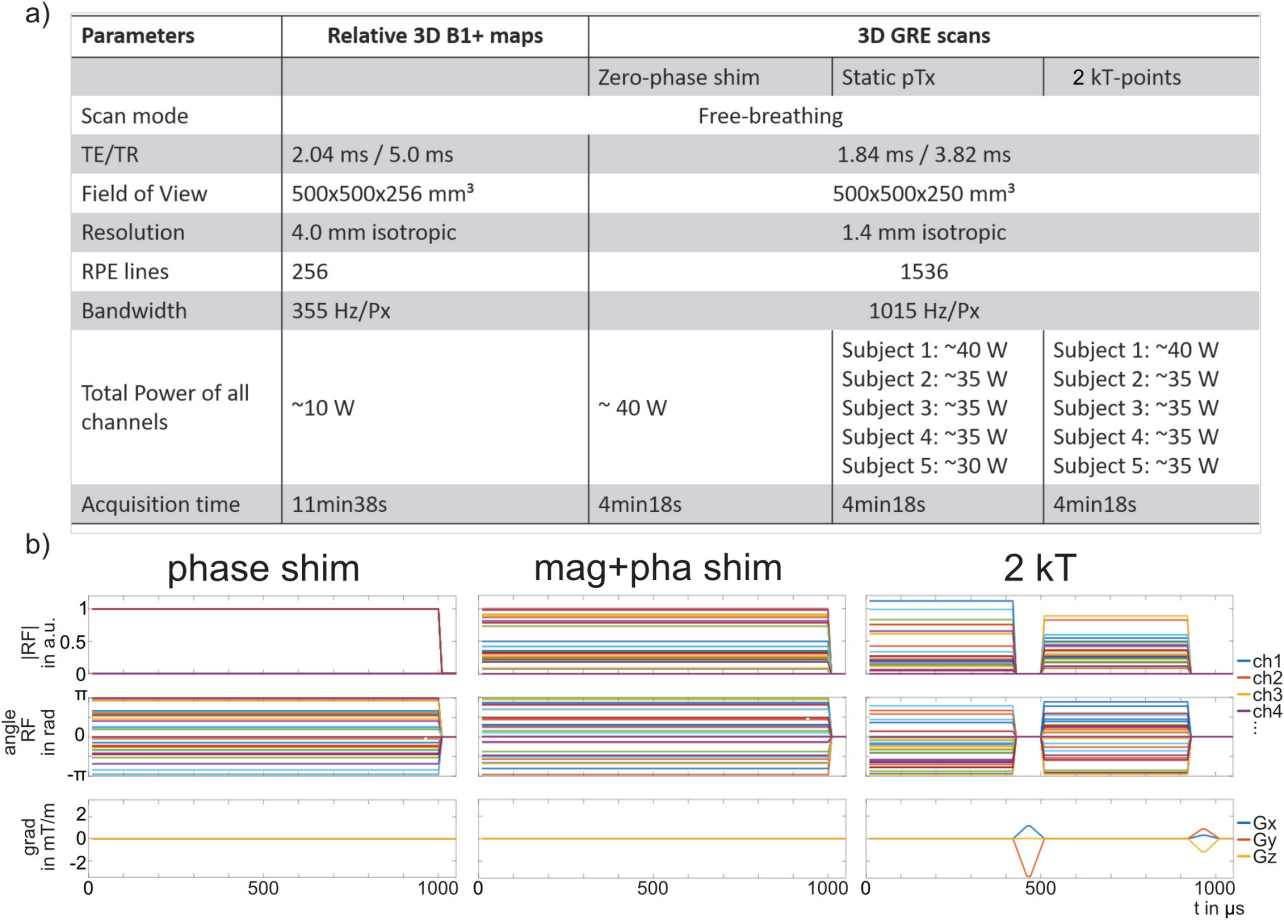
a) Scan parameters for the relative *B*_1_^+^ maps and the GRE acquisitions with zero‐phase shim, static pTx, and two k_T_‐points. Radial phase encoding (RPE) lines are acquired in golden angle scheme [45]. b) Pulse diagram for an example phase shim, magnitude and phase shim and dynamic pTx RF pulse with two k_T_‐points and corresponding gradient trajectories with a total pulse duration of ~1 ms and a blip duration of 80 μs. Magnitudes of channels with instabilities are set to zero.

For each subject, the RF power of the pulses was adjusted to be within the power limits of the safety supervision. The safety supervision operated in power‐controlled mode [41], in which transmit power limits were enforced on a per‐channel basis [42]. The channel‐wise power limits were determined based on simulations and set to 6.6 W per channel averaged over 10 s and 3.3 W per channel averaged over 6 min (RF power measured at the directional couplers). Second‐order *B*_0_ shimming was performed using a vendor‐supplied work‐in‐progress package. According to previous works [29, 43], relative 3D *B*_1_^+^ maps of the liver were obtained for all channels during free‐breathing by a fast estimation technique using a radial phase encoding (RPE) 3D acquisition [44]. A total of 34 measurements (the first measurement with all channels being active with equal magnitudes and phase 0, the second measurement with all channels inactive, and measurements three to 34 with only one channel transmitting, whereas the others are inactive) with a 4‐mm isotropic resolution resulted in a total acquisition time of 11:38 min. Please note the increased scan time compared to previous works [29, 43] due to the larger number of Tx channels. The scan parameters are shown in Figure 2a. During this scan, the subjects performed shallow breathing; thus, the raw data could be reconstructed without respiratory binning [46]. An ROI was manually drawn on the transversal root‐sum‐of‐squares *B*_1_^+^ images on every fourth slice covering the whole liver, which was used for the pulse optimization, whereas the region outside of the ROI was disregarded during the optimization.

Reconstruction of the relative *B*_1_^+^ maps and the RF pulse optimization were performed on a separate workstation (8 cores with 2.3 GHz and 256 GB RAM), and the acquired free‐breathing in vivo gradient recalled echo (GRE) images were reconstructed on a workstation (16 cores with 2.6 GHz and 512 GB RAM).

### 2.3 RF Pulse Optimization

Based on the measured 3D relative *B*_1_^+^ maps, static and k_T_‐point RF pulses with up to six k_T_‐points [27] were calculated to investigate the necessary amount of k_T_‐points for a homogeneous excitation of the liver, aiming to achieve the lowest possible CV at the same RF power level as for phase shimming. Magnitude and phase shimming k_T_‐point is equivalent to phase/magnitude *B*_1_^+^ shimming and is optimized in the ROI by minimizing the cost function that trades off between the coefficient of variation ($\mathrm{CV}=\mathrm{std}\left(|\sum_{\mathrm{ch}=1}^{32}{\hat{B}}_{1,\mathrm{ch}}^{+}b_{\mathrm{ch}}|\right)/\text{mean}\left(\left|\sum_{i=1}^{32}{\hat{B}}_{1,\mathrm{ch}}^{+}b_{\mathrm{ch}}\right|\right)$ and transmit efficiency $\eta =(|\sum_{\mathrm{ch}=1}^{32}{\hat{B}}_{1,\mathrm{ch}}^{+}b_{\mathrm{ch}}$|)/$\left(\left|\sum_{i=1}^{32}{\hat{B}}_{1}^{+}\right|\right)$, where ${\hat{B}}_{1,\mathrm{ch}}^{+}$ are the estimated $B_{1}^{+}$ maps of each channel ch and $b_{\mathrm{ch}}=a_{\mathrm{ch}}e^{i\phi_{\mathrm{ch}}}$ are the complex RF weights with the channel‐dependent magnitudes $a_{\mathrm{ch}}$ and the channel‐dependent phase offsets $\phi_{\mathrm{ch}}$[8, 29, 30]. For phase shimming, the magnitude remains equal across channels. Following previous works [29], k_T_‐point pulses with two to six k_T_‐points were calculated in the small tip angle approximation with interleaved greedy and local optimization methods [47, 48] for each of the subjects to excite the ROI with a nominal 10° FA by solving

(1) $$\min_{b}\frac{1}{2}{\left\|\left|m_{d}\right|-\left|\sum_{\mathrm{ch}=1}^{32}{\hat{B}}_{1,\mathrm{ch}}^{+}A\left(K\right)b_{\mathrm{ch}}\;\right|\right\|}_{\mathrm{ROI}}^{2}+\frac{\beta }{2}{\left\|b_{\mathrm{ch}}\right\|}^{2},$$

where $m_{d}$ is the desired FA pattern, $A\left(K\right)$ the excitation system matrix, and β the regularization parameter [47]. Each optimization was carried out with 100 pseudo‐random starting phases and equal amplitudes. The total pulse duration was fixed to approximately 1 ms to minimize the influence of *B*_0_ [29], with a fixed blip duration of 80 μs; thus, the individual k_T_‐point subpulse duration decreased with increasing number of k_T_‐points (magnitude and phase shimming: 1 ms, 2 k_T_: 420 μs, 3 k_T_: 253 μs, 4 k_T_: 170 μs, 5 k_T_: 120 μs, 6 k_T_: 87 μs).

For the analysis, for magnitude and phase shimming and each k_T_‐point, the regularization parameter β was iterated between 10^−10^ and 10^3^, trading between total RF power and excitation fidelity. The RF power is used as a constraint rather than local SAR, because the safety supervision of the system is presently based on a power constraint that limits the maximum power per channel [42, 49]. For the in vivo application, the updating of the RF regularization parameter was done every 50 iterations as described in Grissom et al. [47] The RF pulse and gradient waveforms are shown in Figure 2b for a representative subject for the phase shim, magnitude and phase shim and for two k_T_‐points.

### 2.4 Analysis

The impact of the number of k_T_‐points on the excitation fidelity within the ROI using the 32‐channel array was examined based on L‐curves. These L‐curves were generated for magnitude and phase shimming and two to six k_T_‐point pulses for each subject by plotting the integrated RF power [50] versus the CV of the FA within the ROI. To allow for direct comparison, the total pulse duration was fixed to 1 ms as stated above (c.f. Figure 2b).

**FIGURE 3:**
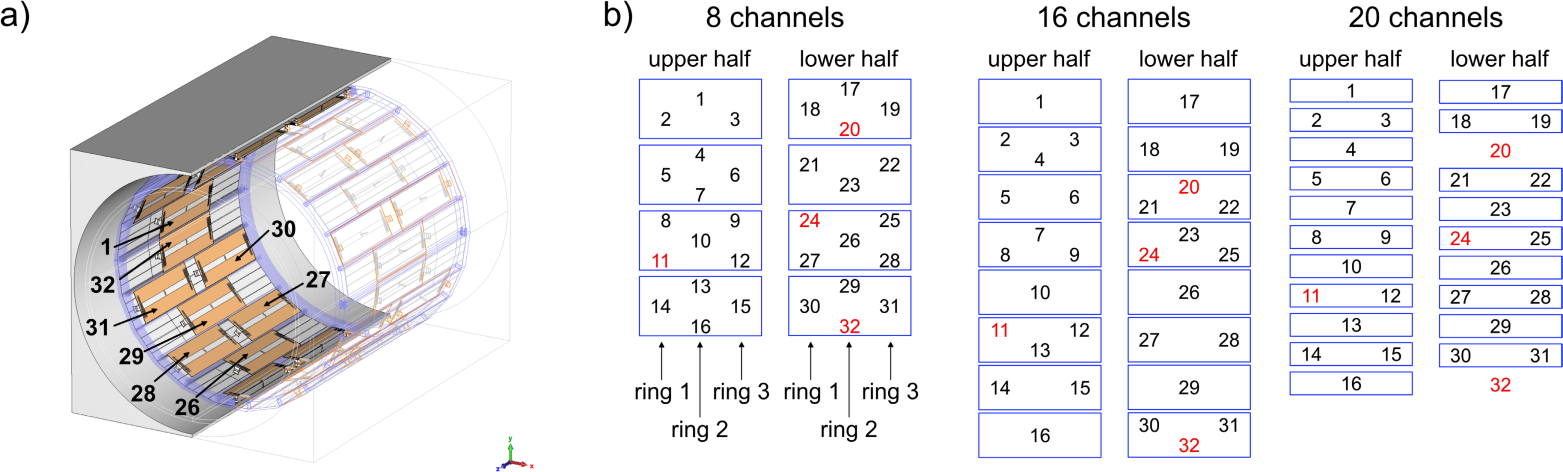
a) Schematic of the 32‐Tx‐channel coil array. The remote array is arranged into three rings, where the two outer rings consist of 10 elements and the inner ring of 12 elements. For symmetrical purposes, the array is split into two halves (element 1–16 and element 17–32) [24, 38]. b) Combination of elements to achieve 8, 16, and 20 Tx channels marked with a blue rectangular. Elements with instabilities are marked in red and are neglected for pulse optimization.

Furthermore, the influence of the number of Tx channels on the resulting excitation fidelity was investigated by generating additional L‐curves for k_T_‐point pulses with one to five k_T_‐points in the first three subjects with i) 8 Tx channels, ii) 16 Tx channels, and iii) 20 Tx channels (Figure 3). For this analysis, elements were grouped as indicated in the blue boxes of Figure 3, with all elements in each group driven with identical phase (zero degrees between the grouped elements). The total available RF power was kept constant across all configurations, and thus, the available per channel power increases with decreasing number of channels.

Additionally, for the same three subjects and all four transmit configurations, L‐curves were generated trading between relative peak local SAR and FA homogeneity (CV). Therefore, the power constraint in Equation 1 was replaced by a relative peak local SAR constraint of the form $\max\left(b_{\mathrm{ch}}^{H}\cdot \text{VOPs}\cdot b_{\mathrm{ch}}\right)$. Virtual observation points (VOPs) [51] for the liver region were derived from four body models (Duke [52], Ella [52], Gustav [53] and Laura [53]).

### 2.5 Validation

To qualitatively validate the predicted FA distributions in vivo, free‐breathing 3D GRE scans were acquired with an RPE acquisition scheme [45] with two k_T_‐point pulses in all volunteers. Additionally, a phase shim, magnitude and phase shim, and a zero‐phase shim were obtained. The scan parameters are shown in Figure 2. The nominal FA for each acquisition was adapted such that the same total RF power was applied for all scans.

## 3 Results

### 3.1 Analysis

**FIGURE 4:**
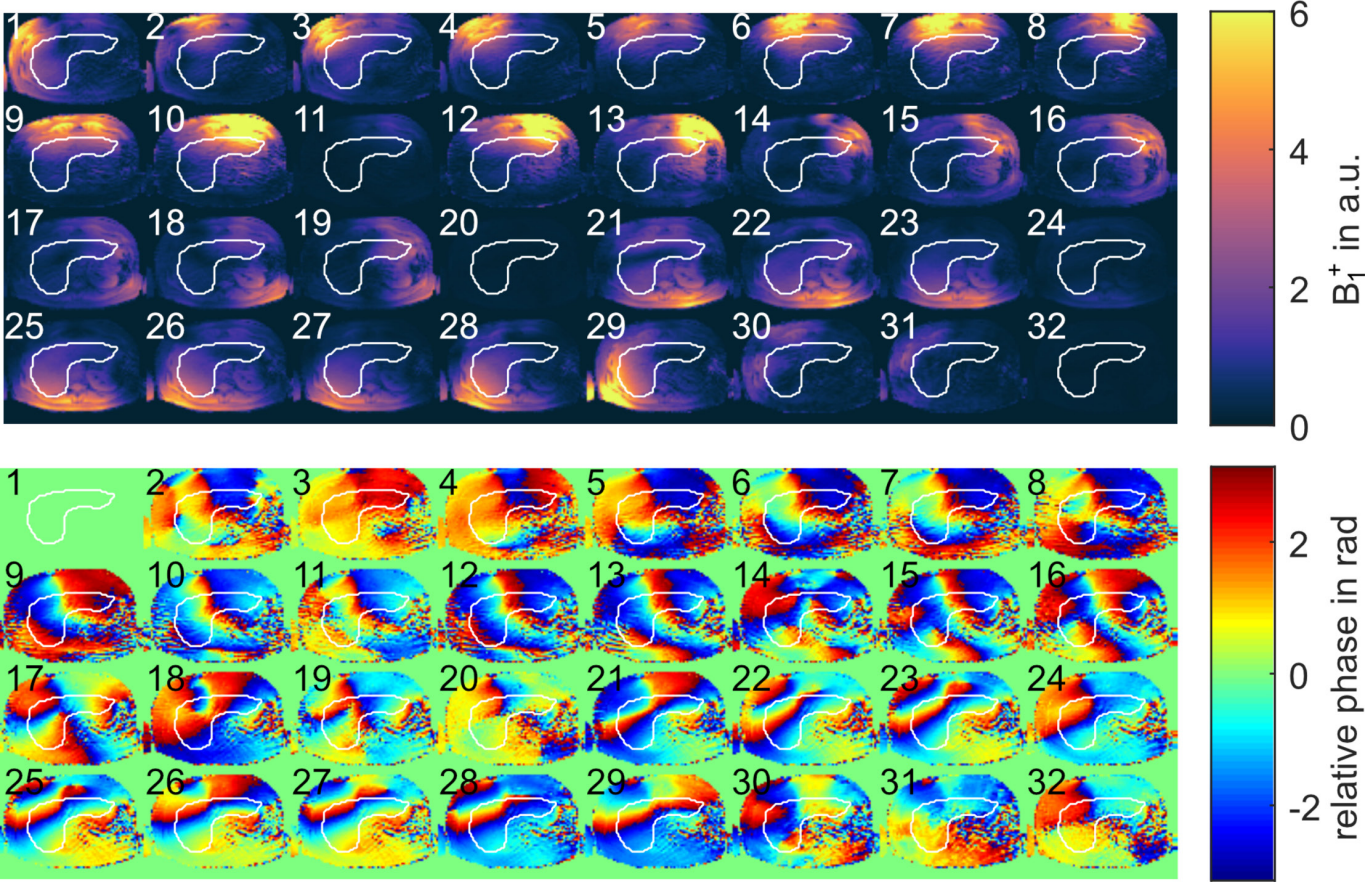
Relative *B*_1_^+^ sensitivity maps for all 32 channels of an example slice through the liver showing a) magnitude and b) relative phase of the channels with respect to Channel 1.

Figure 4 shows relative *B*_1_^+^ magnitude and phase maps and the respective ROI used for optimization of one representative subject in an example transversal slice through the liver. Instabilities of channels 11, 20, 24, and 32 can be seen in the magnitude maps as they have values close to zero. Furthermore, residual artifacts are visible in the magnitude images (i.e., channel 10 in the center of the subject) that result from the undersampling of the RPE.

#### 3.1.1 Comparison With All Channels

L‐curve plots obtained after *B*_1_^+^ shimming and with dynamic pTx pulses using up to six k_T_‐points are shown in Figure 6 for the five subjects and all Tx channels enabled. Phase shimming yields CV values below 15% for four subjects, which indicates FA distributions without dropouts within the ROI, as recently reported for the heart volume at 7 T [54] and as confirmed by Figure 6a for Subject 4. For Subject 5 with the largest body dimension in the anterior–posterior direction, a CV value of 17.9% was obtained.

With magnitude and phase shimming and the same RF power as for phase shimming, the CV can be improved just by a factor of 1.1, and the resulting *B*_1_^+^ predictions improve only slightly (Figure 6a). Applying two k_T_‐points results in a reduction in CV by a factor of 2.6–3.1 versus phase shimming and in maps with only minor remaining FA variations in any subject for the same RF power as for phase shimming.

**FIGURE 5:**
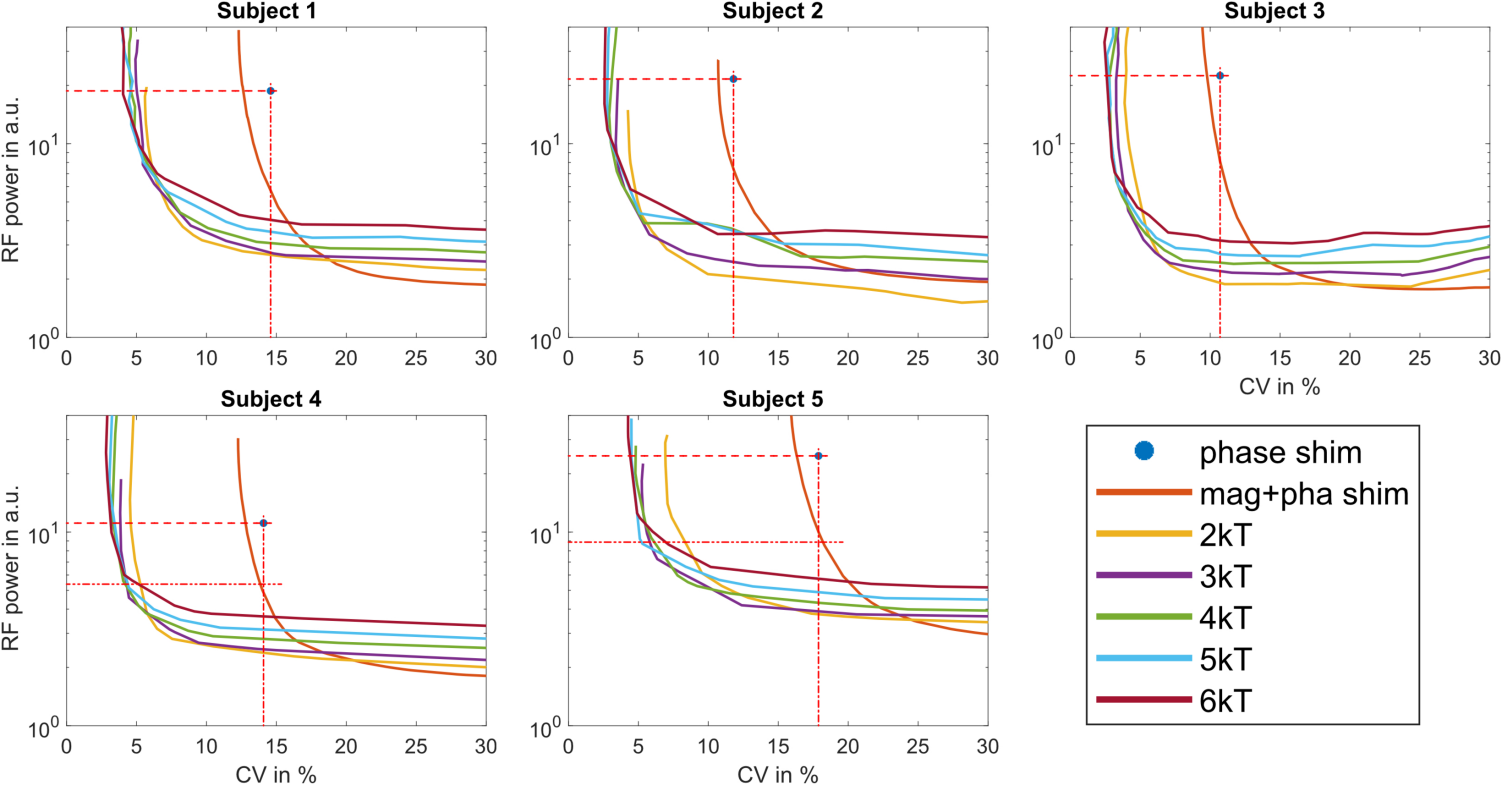
Integrated RF power vs. FA CV for all subjects for magnitude and phase shimming and up to six k_T_‐point pTx pulses. Horizontal dashed lines mark the same RF power as for phase shimming and the vertical dotted line the same CV as for phase shimming. For Subjects 4 and 5, the second horizontal dotted line marks half of the RF power as for phase shimming, representing a more favorable tradeoff between power and homogeneity, that is, a value close to the corner of the L‐curve.

**FIGURE 6:**
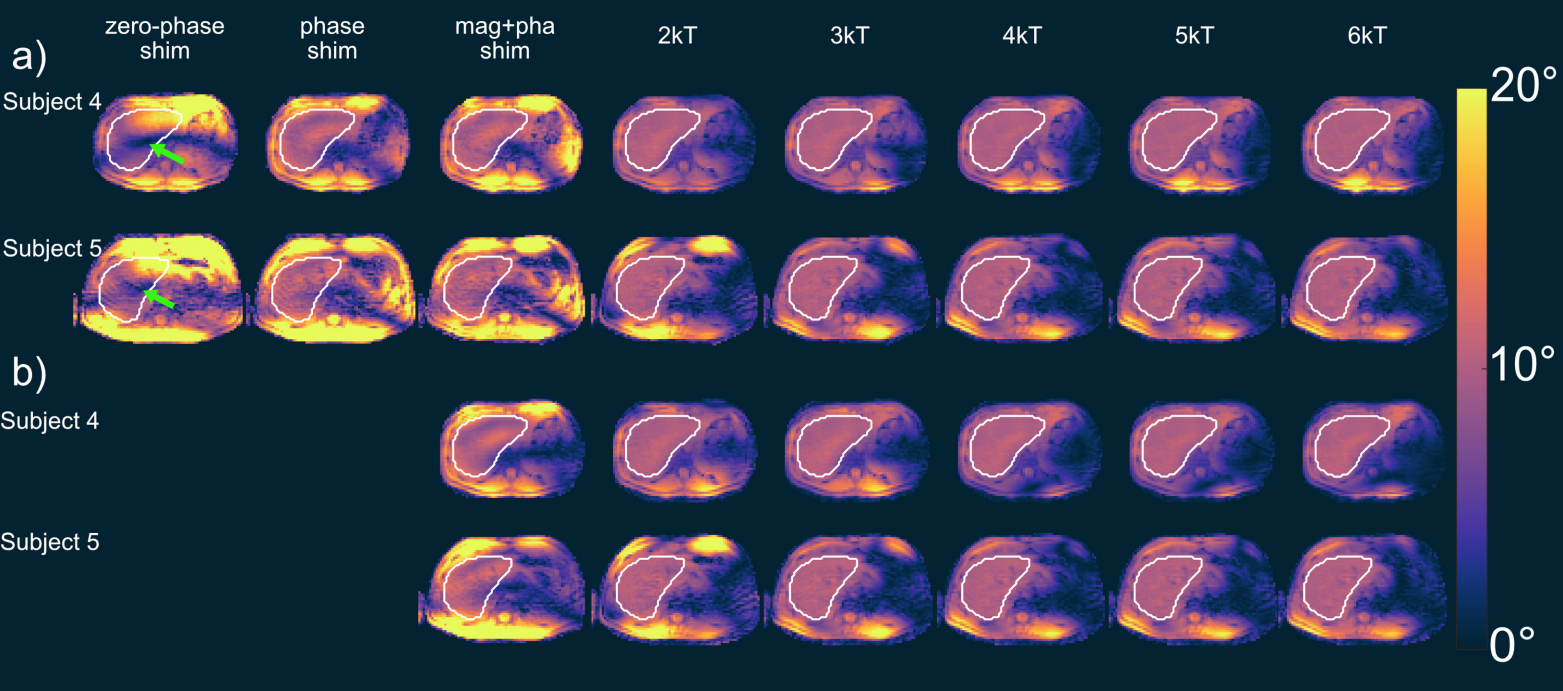
FA predictions for an example transversal slice through the liver for Subjects 4 and 5 with a) the same RF voltage as phase shimming and b) half of the RF power as for phase shimming. FA dropouts are marked with a green arrow in the zero‐phase shim.

A further but less pronounced decrease in CV, which is also qualitatively visible in the FA predictions in Figure 6a, is obtained when the number of k_T_‐points is further increased while maintaining the same RF power. By comparing the different numbers of k_T_‐points with half of the RF power as for phase shimming (lower horizontal dotted line in Figure 5), the more optimal tradeoff between power and homogeneity, that is, a value close to the corner of the L‐curve, can be obtained. In this case, the CV for two k_T_‐points is reduced by a factor of 2.4 for Subject 4 and by a factor of 2.7 for Subject 5 in comparison to magnitude and phase shimming. Again, the homogeneity is only marginally improved by increasing the number of k_T_‐points, as is qualitatively visible in Figure 6b.

As an alternative to reducing the CV, the RF power can be reduced by a factor of 2.2–2.9 by magnitude and phase shimming and by a factor of 4.7–10.1 for two k_T_‐points versus phase shimming for the same CV as for phase shimming. In this case, increasing the number of k_T_‐points does not reduce the RF voltage RMS further. For all five subjects, the choice of two to three k_T_‐points resulted in a good tradeoff between excitation fidelity and RF power (bend of the L‐curve) as neither the CV nor the RF power decrease significantly with a further increase in the number of k_T_‐points.

#### 3.1.2 Comparison of Different Channel Numbers

**FIGURE 7:**
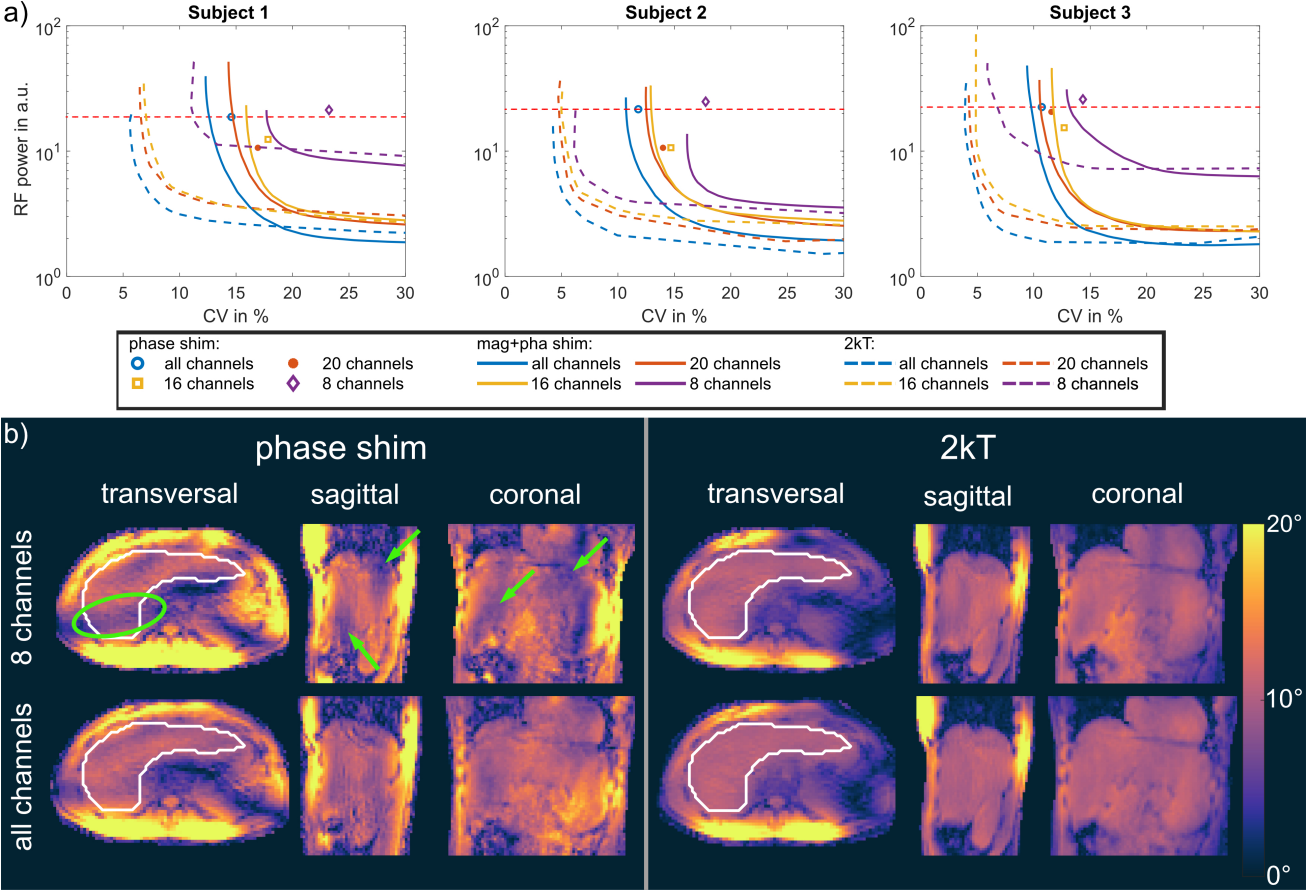
a) L‐curves for Subjects 1, 2, and 3 for 8, 16, 20, and 32 channels. The horizontal dotted line marks the same RF power as for phase shimming with 32 channels. b) FA predictions for Subject 1 comparing 8 and all 32 channels with phase shim and two k_T_‐points. Green arrows and circles mark FA dropouts and variations with 8 channels; these areas show improved homogeneity with 32 channels.

Figure 7a compares the L‐curves obtained with 8, 16, 20, and all 32 Tx channels for three subjects with low (Subject 1), normal (Subject 3), and high (Subject 2) BMI. For all subjects, increasing either the number of Tx channels or the number of k_T_‐points reduces the CV, yielding increased homogeneity. For phase shimming, the CV decreases by a factor of 1.3–1.6 going from 8 channels to 32 channels. By evaluating the homogeneity across different channel numbers while maintaining the RF power used for phase shimming with 32 channels, the CV can be reduced by a factor of 1.08–1.25 across subjects when increasing from 8 to 16 channels for magnitude and phase shimming. For two k_T_‐points, the CV reduction ranges from a factor of 1.25–1.47 for the same channel increase. Further increasing the number of channels to all 32 Tx channels reduces the CV by a factor of 1.16–1.21 for magnitude and phase shimming and 1.17–1.26 for two k_T_‐points.

As expected, comparing 8 channels to 32 gave the largest improvement: A factor of 1.34–1.50 for magnitude and phase shimming and 1.47–2.03 for two k_T_‐points. Furthermore, phase shimming with 32 channels yielded lower CV values than magnitude and phase shimming for 8 and 16 channels. The largest increase was seen in Subject 1, where five k_T_‐points are necessary to achieve similar CV values as two k_T_‐points with 16, 20, or all channels (not shown). Furthermore, phase shimming with 8 channels results in a CV of 23.2%, where a FA dropout can be found in the ROI that is marked with a green arrow (ellipse) in Figure 7b. In this figure, the improvement of all channels in comparison to 8 is also qualitatively visible for phase shimming and for two k_T_‐points. Additionally, the FA dropout that appears in phase shimming with 8 channels is eliminated by using all 32 channels. This improvement by phase shimming, achieved by increasing the number of channels from 8 to 32, is also evident in the in vivo GRE images of Subject 1 shown in Figure S1.

Furthermore, using 32 channels requires less RF power than using 8, 16, or 20 channels to achieve the same level of homogeneity across all subjects. When comparing the required RF power for two k_T_‐point pulses using 16 channels versus 8 channels, a reduction in power by a factor of 1.23–3.11 is observed, depending on the subject. A further comparison between 16 and 32 channels reveals that, for the same target homogeneity (as achieved with phase shimming using 32 channels) and two k_T_‐points, the required power is reduced by an additional factor of 1.38–1.6 when using 32 channels.

Figure S2 presents the L‐curves for 8, 16, 20, and 32 channels in the same three subjects, optimized to tradeoff between relative peak local SAR and FA homogeneity. The dependence of FA homogeneity on both the number of k_T_‐points and the number of channels follows the same trend as observed for L‐curves optimized with the RF power constraint, namely a progressive improvement in homogeneity. Moreover, comparison of the corner of the L‐curve (indicated by the gray arrows) between 8‐ and 32‐channel configurations demonstrates that, for two k_T_‐points, the peak local SAR is reduced by a factor of 1.6–2.5 across all subjects, in addition to the observed improvement in FA homogeneity. Furthermore, while subjects one and three show additional reductions in peak local SAR for two k_T_‐points when increasing the number of channels from 16 to 20 and from 20 to 32, subject 2—who had the highest BMI—does not exhibit further SAR reduction beyond 16 channels.

### 3.2 Experimental Validation

**FIGURE 8:**
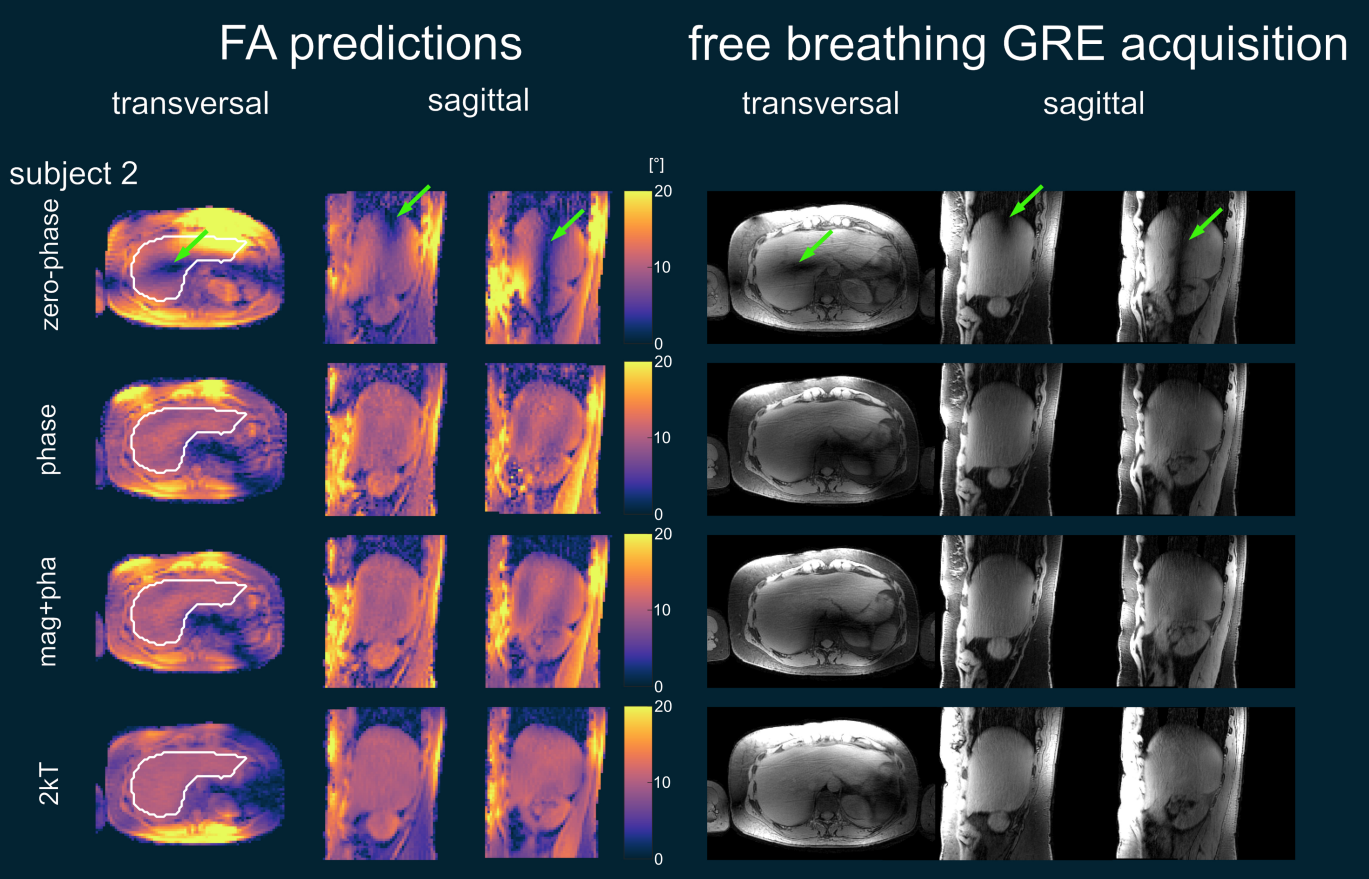
FA predictions and corresponding magnitude images of an example transversal slice and two example sagittal slices through the liver of Subject 2 for zero‐phase shim, phase shim, magnitude and phase shim and for two k_T_‐points. The FA dropout in the zero‐phase shim is marked with a green arrow. The receive profile is not removed.

**FIGURE 9:**
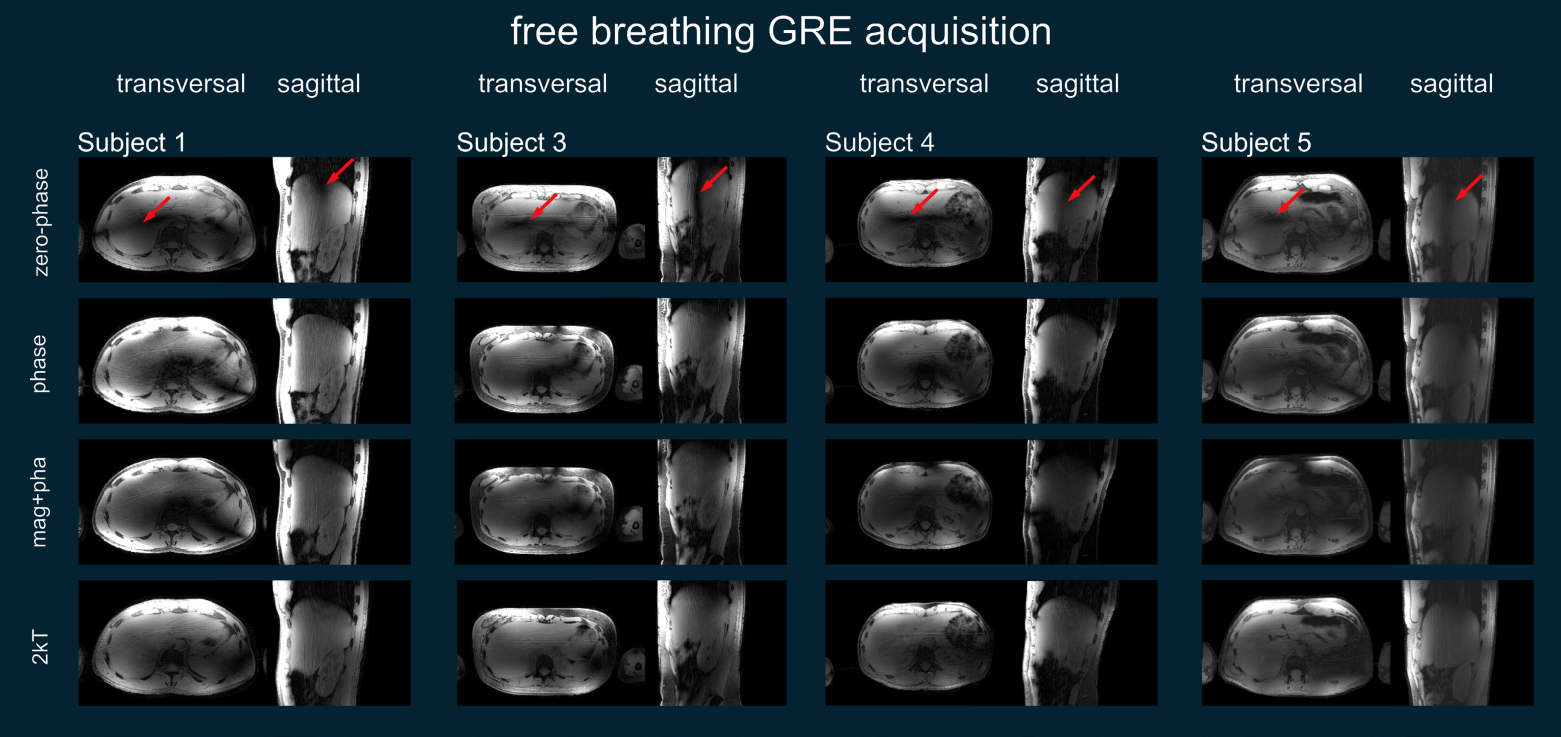
GRE acquisitions for transversal and sagittal orientations of Subjects 1, 3, 4, and 5 for zero‐phase shim, phase shim, magnitude and phase shim and two k_T_‐points. The FA dropout for the zero‐phase shim is marked with a red arrow in all subjects. Note that the receive profile is not removed. In addition, residual streak artifacts are visible that originate from the undersampling of the RPE.

Figure 8 shows the in vivo GRE images of a representative transversal slice and two sagittal slices through the liver using all available channels for Subject 2. The signal magnitudes of these small FA acquisitions match their corresponding predictions. The FA dropouts are clearly visible for the zero‐phase mode in both prediction and measurement. These dropouts are removed using phase shimming or magnitude and phase shimming. It is also visible in the transversal orientation that two k_T_‐points improve the FA homogeneity further in both predictions and the corresponding GRE scans. Figure 9 shows the GRE images for the other subjects in transversal and sagittal orientation and with zero‐phase shim, phase shim, magnitude and phase shim and for two k_T_‐points. The FA dropouts in the zero‐phase shim are marked with a red arrow. For all subjects, no dropouts are left in the case of phase shimming, and magnitude and phase shimming provided a slight improvement in homogeneity compared to phase shimming. However, FA variations are still visible, which are removed when using two k_T_‐points.

## 4 Discussion

In this work, the performance of static and dynamic pTx using k_T_‐point pulses in combination with a remote whole‐body array was evaluated in vivo for human liver MRI at 7 T. An aim of this work was to investigate if the large amount of array elements (32 independent channels) and the larger distance to the body achievable with a remote whole‐body array relative to local coil arrays provide an advantage and, in addition, if the number of k_T_‐points necessary for homogeneous liver excitation can be reduced.

Based on the relative *B*_1_^+^ maps and the FA predictions obtained for different channel counts, this work indeed demonstrates that a higher number of channels is advantageous, corroborating the results of Fiedler et al. [37] that were obtained from simulated *B*_1_^+^ maps. In particular, the work shows that a remote array with 32 Tx channels and two k_T_‐points provides lower CV values as compared to 8 Tx channels and two k_T_‐points, and only by increasing the number of k_T_‐points a similar CV value be achieved for the 8 Tx channels. The improvement in comparison to 16 or 20 channels is less pronounced, although a higher gain might be expected when an even larger ROI such as the whole abdomen is targeted.

The present study further showed that static pTx yields sufficient FA homogeneity for normal‐weight subjects, that is, CV values of below 17.9% were achieved in all subjects in the *B*_1_^+^ predictions and also reflected in the GRE acquisitions. From an application standpoint, static pTx is beneficial as it does not require changing the sequence code and thus it is compatible with existing and widespread clinical sequences and protocols. Therefore, clinical trials and studies investigating liver diseases can directly benefit from static pTx in combination with the remote array [22, 55, 56, 57].

Dynamic pTx using two k_T_‐points improved the homogeneity even further and is a good tradeoff between power and FA homogeneity, as the homogeneity did not increase substantially with more k_T_‐points. In addition, preliminary results [58] showed that, for instance, for a subject with a BMI of 27.1 kg/m^2^ and a body dimension in the anterior–posterior direction of above 23 cm, static pTx is no longer sufficient. For this subject, dynamic pTx using two k_T_‐points improved the CV values to below 13% and delivered acceptable FA homogeneity. In comparison, it has been shown in subjects with a normal BMI that it is challenging to achieve good homogeneity over a large ROI in a single slice [17] or in a large volume [18] when utilizing local arrays in combination with static pTx. Furthermore, it has been shown that 3–4 k_T_‐points delivered a practical tradeoff between nominal FA homogeneity and RF power in the heart [29]. Additionally, a homogeneous excitation in the liver was achieved for five k_T_‐points using a local array [31]. We also acquired three k_T_‐point pulses at the end of each session, which did not show an improvement in homogeneity compared to two k_T_‐points (not shown). The validation matched the prediction in three subjects; however, we noticed deviations in two subjects, which are expected to be caused by motion between the acquisition of the relative *B*_1_^+^ maps and the GRE validation scans.

It is expected that the remote array in combination with static or dynamic pTx will deliver similarly homogeneous excitation patterns for other internal organs and regions with the size of the liver or smaller. Therefore, the remote array might also benefit further clinical studies which need homogeneous excitation across a larger area similar to the liver, for example imaging in the abdominal region to investigate lymph nodes in pancreatic, duodenal, or periampullary adenocarcinoma [59] or for noncontrast enhanced renal angiography of both kidneys [13].

As an alternative to dynamic pTx, TIAMO can be applied for the liver, which also yields sufficient FA homogeneity [25]. In comparison to static or dynamic pTx, however, TIAMO typically requires longer scan times.

As a relatively short total pulse length of approximately 1 ms was chosen, Δ*B*_0_ influences could be minimized [29] so that *B*_0_ maps did not have to be additionally acquired and taken into account for pulse optimization, which reduced the total measurement time and which would therefore also be beneficial for clinical applications.

This work is also subject to limitations. The applied system is a prototype, and during the course of the experiments, we observed instabilities in the RF power of some Tx channels that were apparent as low *B*_1_^+^ magnitude values in the resulting relative *B*_1_^+^ maps. To account for this, the relative *B*_1_^+^ maps were analyzed before each session, and the channels with low *B*_1_^+^ amplitude were neglected for pulse optimization, which resulted in 28 available channels for all measurements. Nevertheless, despite four missing Tx channels, this work could demonstrate the benefit of the remote coil array in combination with a much higher number of Tx channels than the standard 8 or 16 channels of commercial pTx systems for liver applications.

Furthermore, the use of local Rx arrays for relative *B*_1_^+^ mapping was necessary to increase SNR and improve the quality of the resulting maps, which may increase the bias of the relative *B*_1_^+^ mapping approach [44]. Nevertheless, the resulting FA predictions and the signal magnitudes of the small FA acquisitions match, suggesting that the influence is limited. With regard to the two local mechanically rigid Rx arrays, it should also be mentioned that the experimental setup (Figure 1) was far from being ergonomic. A more flexible anterior Rx array design would provide increased subject comfort and would also place the individual RF receive loop elements closer to the body surface for better Rx performance.

While it has been demonstrated for several different applications that local SAR constraints can be integrated into the pulse optimization to prevent high local SAR maxima [60, 61, 62, 63], the present work uses the power‐controlled mode [42], a per channel RF power constraint instead of a VOP‐based constraint for in vivo application. The rationale behind this constraint is that the implemented safety supervision takes only the forward RF power into account [42, 64]. A power‐based safety supervision is technically easier to implement in terms of hardware and software for 32 channels than a SAR supervision based on VOP [65, 66], as only half of the data (only the magnitude and not the phase of the signal) is supervised, and the data can be further compressed by averaging the magnitudes of each channel over a short time period. However, this approach is more conservative, permitting less RF power compared to a full phase‐based supervision. Because of decreasing absolute FA in the center of the body with increasing BMI and increasing anterior–posterior body dimensions, the image quality of the relative *B*_1_^+^ maps decreased [58]. With recent improvements in VOP calculation and VOP compression algorithms [51, 67, 68] and the improvement in GPU power, a more precise and less conservative VOP‐based real‐time SAR supervision is feasible [69], which would enable higher FA for channel‐wise *B*_1_^+^ mapping and facilitate the optimization and use of RF pulse designs with a local SAR constraint for in vivo application [60].

As for all subject‐tailored pTx applications, another limitation of this work lies in the need to map the *B*_1_^+^ sensitivity maps of each individual Tx channel. Because of the 4‐fold higher channel count as compared to most pTx systems, the time to map the relative *B*_1_^+^ maps required more than 10 min, which is not acceptable for most clinical applications. However, this extended scan time does not reflect the minimal data required for pulse calculation. Because the smooth spatial variation of the *B*₁^+^ field, high spatial resolution of 4‐mm isotropic is not essential for RF pulse design, and faster acquisitions yielding lower‐resolution *B*_1_^+^ maps of > 10‐mm isotropic are typically sufficient [70]. In this context, very fast *B*_1_^+^ mapping approaches using deep learning based methods [71, 72] might be favorable, because they have the potential to reduce the *B*_1_^+^ mapping duration to a few seconds. As an alternative, calibration free universal dynamic pTx RF pulses could be used, which do not require subject‐specific *B*_1_^+^ maps to be acquired in each subject [54, 73, 74]. However, universal solutions typically provide reduced homogeneity as compared to subject‐tailored pulses.

## 5 Conclusions

This work demonstrates that FA homogeneity of the entire liver can be improved using a 32‐Tx‐channel remote whole‐body array for static pTx, while 2 k_T_‐points achieve similar excitation homogeneity to an 8‐channel system using five k_T_‐points. Moreover, static pTx delivers sufficient FA homogeneity in normal BMI subjects throughout the liver. Dynamic pulses with two k_T_‐points offer a good tradeoff between power and FA fidelity for 3D liver applications in the low FA regime for all subjects, including those with higher BMI.

## Author Contributions

J.A.G.: conceptualization, formal analysis, investigation, methodology, software, validation, writing – original draft preparation, writing – review and editing; C.S.A.: conceptualization, methodology, software, supervision, writing – review and editing; S.D.: resources, software, writing – review and editing; S.O.: resources, writing – review and editing; T.M.F.: resources, writing – review and editing; S.S.: methodology, writing–review and editing; C.S.: investigation, writing–review and editing; H.H.Q.: resources, writing–review and editing; A.M.N.: supervision, writing–review and editing; M.E.L.: funding acquisition, project administration, resources, supervision, writing – review and editing; S.S.: conceptualization, funding acquisition, project administration, resources, supervision, validation, writing – original draft preparation, writing – review and editing.

## Supporting information


**Figure S1:** In vivo acquisition of Subject 1 with phase shimming. The top row shows the phase shim acquired with all 32 channels (CV of optimization = 17.6%) and the bottom row with 8 channels (CV of optimization = 24.3%).
**Figure S2:** L‐curves for Subjects 1, 2, and 3 for 8, 16, 20, and 32 channels, optimized to minimize the relative peak local SAR using 157 VOPs. The gray arrow indicates the bend of each L‐curve.

## Data Availability

The following data will be provided upon request: relative 3D B1+ maps acquired using the remote 32‐channel transmit array for all five subjects, without respiratory binning.
