# Supplementary material for: In Vivo 3D Liver Imaging at 7 T Using kT‐Point pTx Pulses and a 32‐Tx‐Channel Whole‐Body Radiofrequency Coil Array
Source: NMR Biomed. 2025 Oct 28;38(12):e70170. doi: 10.1002/nbm.70170 (PMC12560210; doi:10.1002/nbm.70170)
Supplement: Supplementary file 1 — Figure S1: In vivo acquisition of Subject 1 with phase shimming. The top row shows the phase shim acquired with all 32 channels (CV of optimization = 17.6%) and the bottom row with 8 channels (CV of optimization = 24.3%). Figure S2: L‐curves for Subjects 1, 2, and 3 for 8, 16, 20, and 32 channels, optimized to minimize the relative peak local SAR using 157 VOPs. The gray arrow indicates the bend of each L‐curve. [file NBM-38-e70170-s001.docx]

Supplementary Material


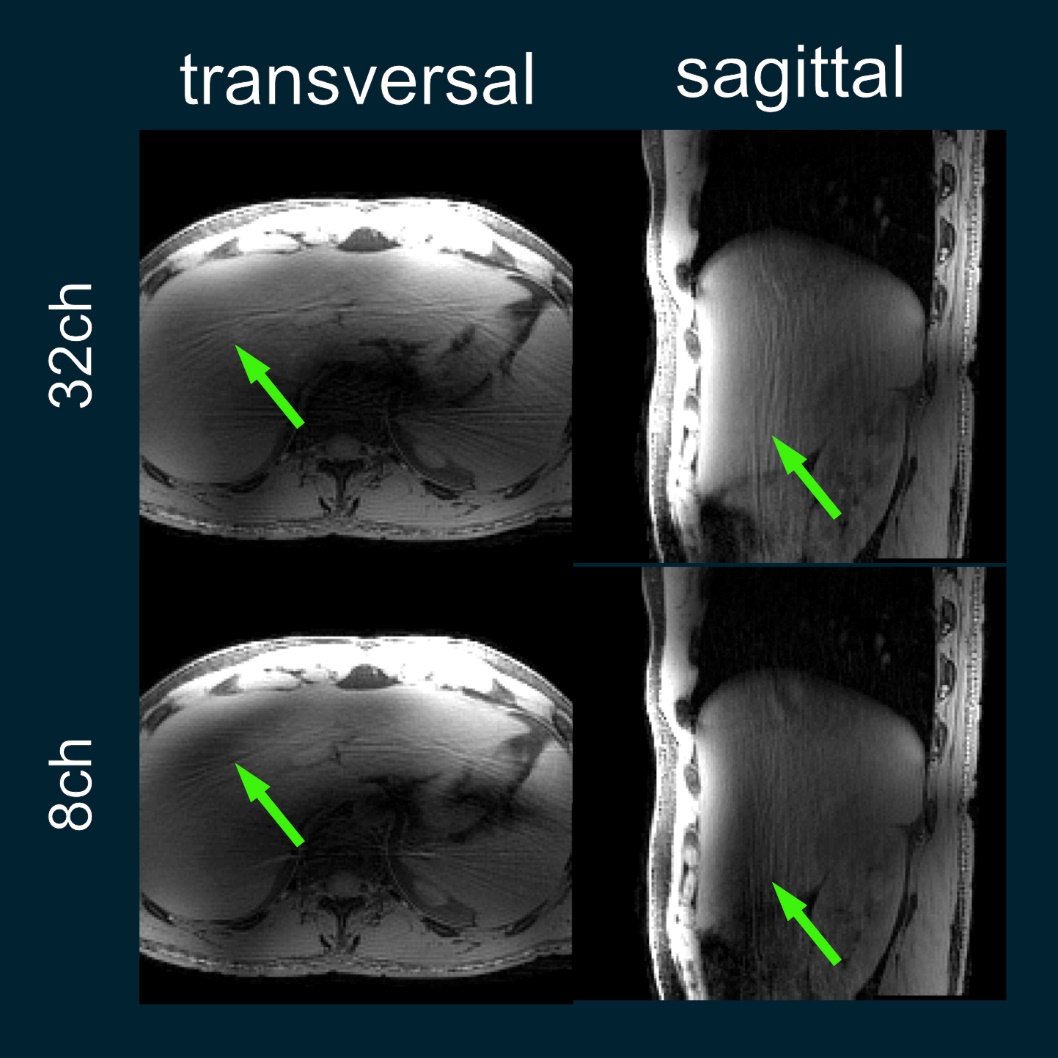


Figure S1: In-vivo acquisition of Subject 1 with phase shimming. The top row shows the phase shim acquired with all 32 channels (CV of optimization = 17.6%) and the bottom row with 8 channels (CV of optimization = 24.3%).


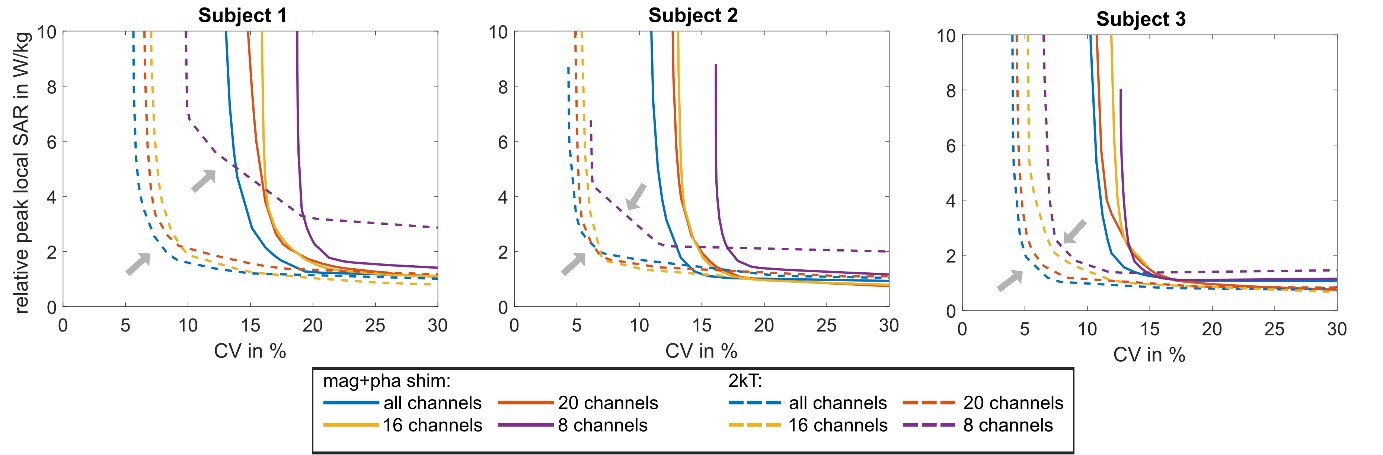


Figure S2: L-curves for Subjects 1, 2, and 3 for 8, 16, 20, and 32 channels, optimized to minimize the relative peak local SAR using 157 VOPs. The gray arrow indicates the bend of each L-curve.
